# Impact of Genomic Prediction Model, Selection Intensity, and Breeding Strategy on the Long-Term Genetic Gain and Genetic Erosion in Soybean Breeding

**DOI:** 10.3389/fgene.2021.637133

**Published:** 2021-09-01

**Authors:** Éder David Borges da Silva, Alencar Xavier, Marcos Ventura Faria

**Affiliations:** ^1^Department of Agronomy, Universidade Estadual do Centro-Oeste, Guarapuava, Brazil; ^2^Department of Biostatistics, Corteva Agriscience^TM^, Johnston, IA, United States; ^3^Department of Agronomy, Purdue University, West Lafayette, IN, United States

**Keywords:** long-term gains, soybean breeding, genomic selections, selection intensity, genomic prediction

## Abstract

Genomic-assisted breeding has become an important tool in soybean breeding. However, the impact of different genomic selection (GS) approaches on short- and long-term gains is not well understood. Such gains are conditional on the breeding design and may vary with a combination of the prediction model, family size, selection strategies, and selection intensity. To address these open questions, we evaluated various scenarios through a simulated closed soybean breeding program over 200 breeding cycles. Genomic prediction was performed using genomic best linear unbiased prediction (GBLUP), Bayesian methods, and random forest, benchmarked against selection on phenotypic values, true breeding values (TBV), and random selection. Breeding strategies included selections within family (WF), across family (AF), and within pre-selected families (WPSF), with selection intensities of 2.5, 5.0, 7.5, and 10.0%. Selections were performed at the F4 generation, where individuals were phenotyped and genotyped with a 6K single nucleotide polymorphism (SNP) array. Initial genetic parameters for the simulation were estimated from the SoyNAM population. WF selections provided the most significant long-term genetic gains. GBLUP and Bayesian methods outperformed random forest and provided most of the genetic gains within the first 100 generations, being outperformed by phenotypic selection after generation 100. All methods provided similar performances under WPSF selections. A faster decay in genetic variance was observed when individuals were selected AF and WPSF, as 80% of the genetic variance was depleted within 28–58 cycles, whereas WF selections preserved the variance up to cycle 184. Surprisingly, the selection intensity had less impact on long-term gains than did the breeding strategies. The study supports that genetic gains can be optimized in the long term with specific combinations of prediction models, family size, selection strategies, and selection intensity. A combination of strategies may be necessary for balancing the short-, medium-, and long-term genetic gains in breeding programs while preserving the genetic variance.

## Introduction

Soybean [*Glycine max* (L.)] is the most important source of protein for animal feed and an important source of oil for human consumption, biofuel, and other industrial applications. Soybeans are cultivated globally, and the largest producers include Brazil, United States, Argentina, Paraguay, and China (FAO, 2021). Soybeans are bred for several traits, but grain yield is considered as the most important.

Genome-wide prediction is a key tool in soybean breeding. It is utilized for faster and more accurate selection of superior individuals (Meuwissen et al., 2001). Methodologically, genomic models recreate the framework utilized for pedigree analysis, but using genomic relationships instead (VanRaden, 2008; Habier et al., 2011; VanRaden et al., 2011). Other factors that may have contributed to the increasing adoption of genomic selection (GS) in plants include the decreasing cost of genotyping and the availability of software tools and computing power to analyze large datasets.

Studies involving GS in plants have been mostly focused on prediction for advancement purposes, hence restricted to the evaluation of genetic gain within a single generation (Schmutz et al., 2010; Sonah et al., 2013; Jarquin et al., 2016; Xavier et al., 2016, 2018a,b; Diers et al., 2018; Smallwood et al., 2019). Studies of long-term gains based on GS are expensive and time-consuming; consequently, the literature is scarce (Wray and Goddard, 1994; Goddard, 2009; Yabe et al., 2016; Gorjanc et al., 2018; Allier et al., 2019a). In addition, evaluation with real data from breeding programs faces additional challenges, such as the ongoing changes in breeding pipelines driven by business decisions, changes in the genotyping technology, and annual changes in resources. Conversely, the deployment of simulations has become an instrumental decision tool in plant breeding. It enables the assessment of genetic gain under different scenarios. In part, the increasing popularity of simulations is due to the quantity and flexibility of software made available (Faux et al., 2016; Pook et al., 2019; Toledo et al., 2019). For instance, breeders are now capable of simulating entire breeding programs with the intent of tuning the breeding parameters to maximize genetic gains in the short and long term (Hickey et al., 2014; Gorjanc et al., 2018), along with the best allocation of resources for a given budget.

By assessing predictive models and contrasting selection strategies, this study envisioned analyzing the influence of a set of variables on long-term genetic gains based on a simulated soybean breeding program and providing insight into the best practices for optimizing genetic gains.

## Materials and Methods

### Simulated Populational Parameters

The founder breeding population contained 200 individuals. Those were simulated based on the genomic parameters using the Markovian Coalescent Simulator (MaCS; Chen et al., 2009), which recreates the evolutionary process with multiple cycles of drift, mutation, and selection. The genomic parameters for the simulations reproduce the soybean genome with detailed information (Schmutz et al., 2010). We considered a genetic map architecture of 20 chromosomes with 115 cM average length, which collectively spanned 950 Mb. For each chromosome, 1,000 segregating sites were assigned.

Our study focused on the simulation of grain yield (in tons per hectare) as the primary trait of interest. The genetic architecture of the simulated trait was assumed to be infinitesimal with 70% of all segregating sites, which were not necessarily utilized as markers, having a non-zero effect sampled from a normal distribution. The genotype-by-environment variance provided a non-heritable variation attributed to the season. Residual variance remained constant throughout the simulation, causing a reduction in heritability overtime as the genetic variance decreased. Simulations began assuming an average yield of 3.00 t ha^–1^. The function *addTraitAEG* from the AlphaSimR package was utilized for the simulation of the phenotypic values. All simulation code is available on GitHub.^1^

Additive genetic effects, genotype-by-environment interaction, and residuals were simulated from Gaussian distribution using variance components estimated from the SoyNAM dataset (Diers et al., 2018; Xavier et al., 2018a) as $\sigma_{\text{a}}^{2}=25$, $\sigma_{\text{G}\times \text{E}}^{2}=49$, $\sigma_{e}^{2}=121$, and *h*^2^ = 0.12. The parameter estimation from the SoyNAM dataset was based on a multivariate genomic best linear unbiased prediction (GBLUP) model with unstructured genetic covariance and diagonal residual covariance, fitting grain yield from all 18 environments as response variables and using as explanatory variables the overall mean (fixed) and a polygenic term (random). The final estimates of the variance components for $\sigma_{\text{a}}^{2}$, $\sigma_{e}^{2}$, and *h*^2^ were obtained as averages across the 18 environments, whereas $\sigma_{\text{G}\times \text{E}}^{2}$ was computed as the average off-diagonal of the variance–covariance matrix.

**FIGURE 1:**
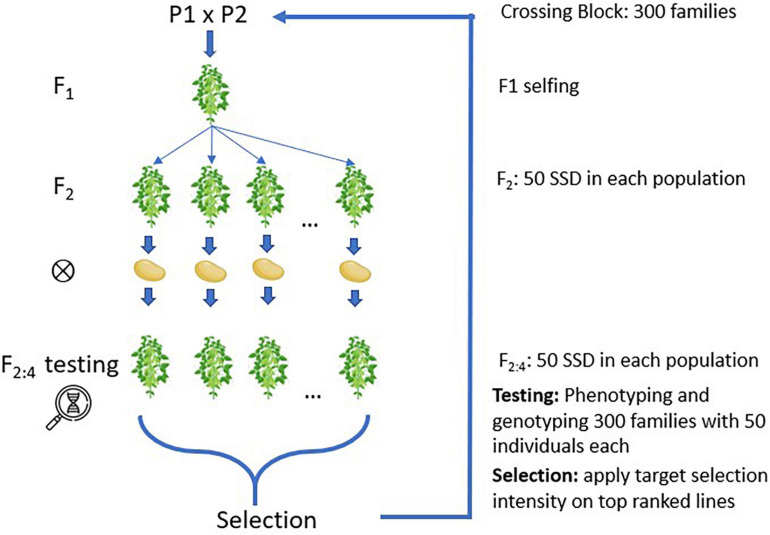
Simulated families created and inbreeding using single seed descent (SSD).

The main simulation settings followed a soybean breeding program with 300 families per cycle and with 50 individuals per family, producing a total of 15,000 individuals per cycle. After crossing, the populations were inbred *via* single seed descendent (SSD) until F_2:4_, as shown in Figure 1, where lines were evaluated in field trials and genotyped with a single nucleotide polymorphism (SNP) array similar to the Soybean 6K SNP chip (Akond et al., 2013). Individuals were then selected to become parents of the upcoming breeding cycle using the phenotypic and genotypic information. The calibration of genomic prediction leveraged data from the previous three breeding cycles, thus leveraging information from up to 45,000 individuals per model. The processes of selecting and crossing were repeated for 200 cycles to capture the theoretical plateau of genetic gains across all simulated parameters. Each breeding scenario was reproduced 60 times with different computational random seeds.

A second simulation with 100 breeding cycles was performed with varying numbers of families and offspring, where five combinations that use the same number of resources were chosen—300 × 50, 250 × 60, 200 × 75, 150 × 100, and 100 × 150—where the combinations correspond to the number of families and individuals per family, respectively. Each breeding scenario was reproduced 45 times with different random seeds.

Genotypic and phenotypic data were simulated with the R package AlphaSimR (Gaynor et al., 2020), reproducing the previous methodological framework (Faux et al., 2016). The software was utilized to simulate the founder population, perform selection, fingerprint individuals with the specified SNP chip, make crosses, generate offspring, inbred individuals, and simulate phenotypic values. All simulations and subsequent statistical analyses of the results were performed using R software (R Core Team, 2020). The code was run in parallel by distributing the multiple breeding scenarios over 960 cores, requiring approximately 10 h of computation per run. The R package doParallel (Ooi et al., 2019) was utilized to parallelize the runs.

### Evaluation of Simulated Scenarios

**FIGURE 2:**
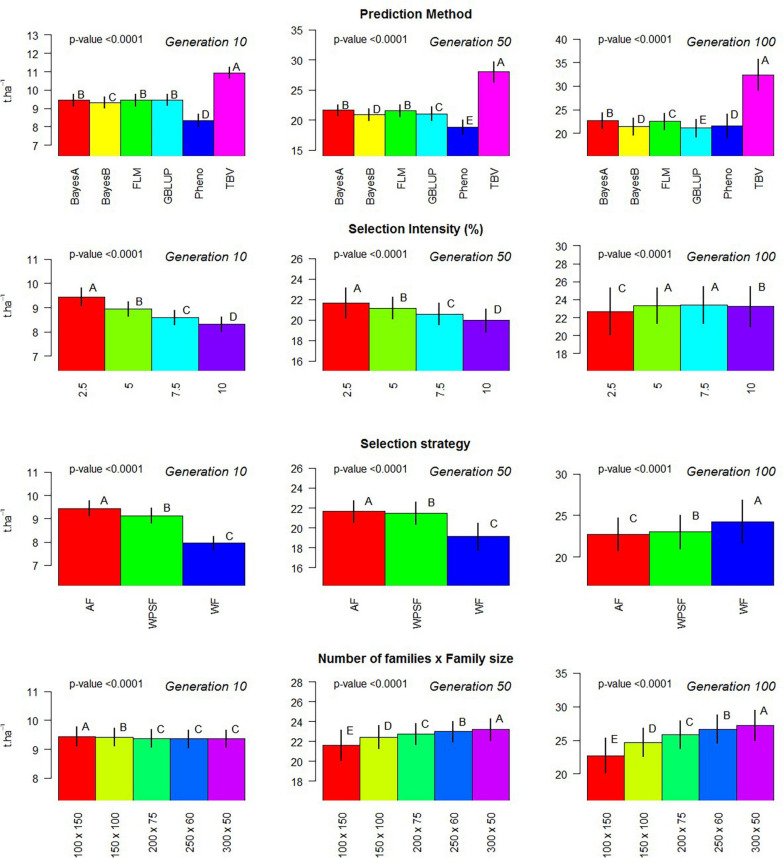
Evaluation of individual factors on population means. Multiple comparison test: Capital letters indicate difference in means across factor with Tukey’s range test with 5% alpha level contrasting the levels of each factor (prediction method, selection intensity, balance between population size and family size, and breeding strategy) on generations 10, 50, and 100.

Evaluation of the breeding strategies, selection intensities, and selection models was based on previous studies (Daetwyler et al., 2013). The evaluation criteria included the population mean across breeding cycles, genetic variance, and accuracy. Analyses were performed within a generation, combining the data from the repeated simulation runs. The statistical model for the analysis of simulated data was the following:

$$y=1\mu +{\text{X}}_{\text{m}}m+{\text{X}}_{\text{s}}s+{\text{X}}_{\text{i}}i+{\text{X}}_{\text{p}}p+\varepsilon$$

where *y* is the vector of the random variable of the simulated population; μ is the model intercept; **X** represents the incidence matrix, which is further divided to accommodate the three factors under evaluation (**X**_m_, **X**_s_, **X**_i_, and **X**_p_); *m* for the selection model; *s* for the breeding strategy; *i* for the selection intensity; *p* for the population design, as combinations of the number of families and individuals per family; and ε is the vector of residuals, assumed to be distributed as $\varepsilon ∼N\left(0,I\sigma_{\varepsilon }^{2}\right)$. The statistical test of multiple comparison was based on Tukey’s range test with 5% probability of error fit using the built-in R function TukeyHSD. This model was used to generate Figure 2.

### Selection Models

The following selection models are evaluated: (1) True breeding values (*TBV*)—true breeding value, which serves as the upper limit of the achievable prediction power; (2) *Random*—random selection of individual, as the worst-case scenario; (3) *Pheno*—phenotypic-based selection without the use of genomic information; (4) *GBLUP*—the genomic best linear unbiased predictor fitted with REML (restricted maximum likelihood) variance components (Nejati-Javaremi et al., 1997; Habier et al., 2007); (5) *BayesA*—Bayesian shrinkage regression that assigns a *t* prior to marker effects (Meuwissen et al., 2001); (6) *BayesB*—an extension of BayesA with variable selection (Meuwissen et al., 2001); (7) *FLM*—fast Laplace model (Xavier, 2019), an empirical Bayes model with a double exponential prior for marker effects; and (8) *RF*—random forest regression (Breiman, 2001), a common machine learning procedure based on bootstrapping aggregation of multiple decision trees. The models GBLUP, BayesA, BayesB, and FLM were fitted using the R package bWGR (Xavier et al., 2019) and solved *via* expectation–maximization (EM). The model RF was fitted using the R package ranger (Wright et al., 2020) with default settings.

As a brief description of the GS model, these models in function on genomic information can be written in terms of the linear model:

y=Xb+f(M)+ε

where *y* is the vector of phenotypic values; **X** is the incidence matrix of the environment term treated as a fixed effect; *b* is a vector of environmental means; *f* (**M**) is the function of markers that describe the genetic merit of individuals; and ε is a random vector of residuals, assumed to be distributed as $\varepsilon ∼N\left(0,\text{I}\sigma_{\varepsilon }^{2}\right)$. The genetic function of markers, *f* (**M**), varied from model to model. For GBLUP, BayesA, and FLM, the function was linear and the marker effects were strictly additive; thus, the function of markers was *f* (**M**) = **M**β. The distinction of the models was the prior assigned to the distribution of marker effects, being normal for GBLUP, distributed as Student’s *t* for BayesA, and distributed as a double exponential for FLM. The function describing BayesB was *f* (**M**) = **M**βγ, which is also linear, but with a variable selection term (γ) that caused further shrinkage to the Student’s *t* prior assigned to the marker effects. The only non-linear model under evaluation was random forest, in which case the genetic function is a linear ensemble of multiple independent regression trees (*T*): *f* (**M**) = *n*^−1^∑*T* (*m* ∈ **M**).

### Breeding Strategy

The breeding strategies were based on soybean breeding designs previously described in the literature (Backes et al., 2003; Sebastian et al., 2010; de Cássia Pereira et al., 2017; da Silva et al., 2018; Smallwood et al., 2019). The following approaches were considered in this study:

*AF*: across-family selection. Genotypes are selected across families based on their estimated genetic merit, without regard for their family structure or any constraint for selecting multiple individuals from the same pedigree.

*WF*: within-family selection. In this strategy, all families were equally represented in the advancements. The best genotypes from each family are selected to become parents in the upcoming generations.

*WPSF*: within the pre-selected family. This strategy comprises two steps. Firstly, the family level selection is performed to identify the best-performing families (top 30%). Secondly, the selection of individuals occurs within the family. With fewer families to select from, more individuals per family will be parenting the upcoming generation compared to WF.

### Selection Intensity

Four levels of selection intensity were considered: 2.5, 5.0, 7.5, and 10.0%. These values represent the percentages of individuals selected to be used as parents of the next generation. The selection of parental combinations was performed at random; thus, it is possible that not all selected individuals served as parents.

## Results

### Genetic Gains

**FIGURE 3:**
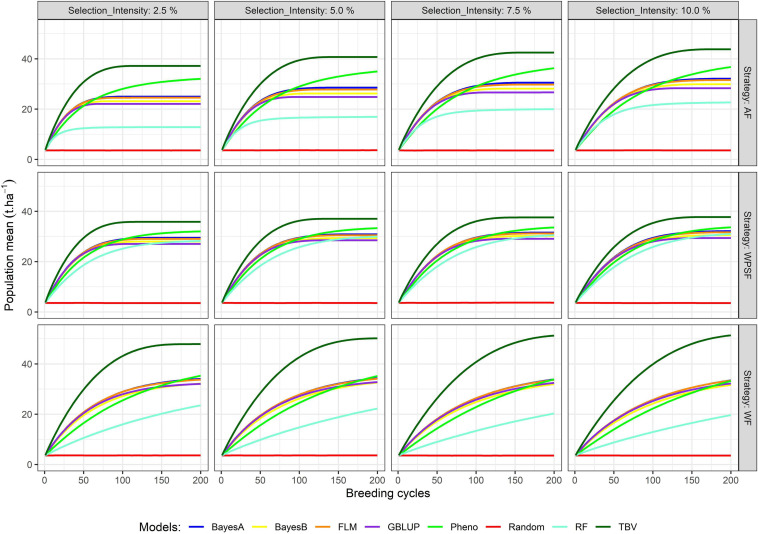
Population means across 200 breeding cycles. Colors correspond to the different selection methods, grid columns represent the selection intensity (2.5, 5, 7.5, and 10%), and grid rows represent the breeding strategies, where individuals were selected across family (AF), within pre-selected families (WPSF), or within-family (WF).

The simulation results presented in Figure 3 summarize the population means over the course of 200 cycles. Supplementary Table 1 provides the population means for all combinations of treatments under evaluation in breeding cycles 10, 100, and 200. Across all scenarios, the population mean of random selection is anchored at the starting point. Selection of TBV represents the upper boundary of each scenario; hence, these are particularly useful to contrast the potential of the different scenarios. The highest long-term population means from selection on TBV occurred WF with loose selection intensities (7.5–10%). Genetic gains were generally closer to those from TBV when selections were performed WPSF.

Phenotypic selection outperformed GS over the course of 200 breeding cycles. Selection using random forest provided poor predictive performance in all scenarios, possibly due to the non-additive nature of the regression trees fitting a strictly additive genetic architecture. All linear genomic models (BayesA, BayesB, FLM, and GBLUP) provided similar outcomes. When conditioning for all other varying parameters, BayesA and FLM were the best-performing models within the first 100 breeding cycles (Figure 2).

After 10 cycles of selection, the highest gains were attained at the highest selection intensity (2.5%), which characterizes the short-term gain benefit from a higher selection pressure while the genetic variance is still abundant. After 100 breeding cycles, the genetic gains are affected by the combination of selection intensity and breeding strategy. For example, selection performed AF using BayesA provided the highest gains with a selection intensity of 10%, whereas, under WF, the highest gains occurred with a selection intensity of 2.5%. Such discrepancy is attributed to the amount of genetic variance left for long-term selection.

The highest long-term gains were reached when selections were performed WF. The maximum attainable, as benchmarked by selection upon TBV, resulted in a grain yield of 54 t ha^–1^ (WF), being 35% higher than AF selections and 46% higher than WPSF (Supplementary Table 2). The overall trend for long-term gains using GS followed the order WF > WPSF > AF. When the selections were based on phenotypic values, the genetic gains outpaced the GS run for all strategies (AF, WPSF, and WF), whereas that was not observed within the first 100 cycles (Figure 1). In fact, phenotypic selection WF was the third highest performing model, behind AF and WF selections performed on TBVs. The impact of each factor on the prediction accuracy over 200 breeding cycles is provided in Supplementary Figure 1.

Figure 2 summarizes the results of the simulation performed within 100 cycles, where different family sizes were an additional variable under evaluation. Within 10 breeding cycles, the scenario of 100 families with 150 individuals displayed the highest average, although the differences were negligible. Over the course of 50 and 100 breeding cycles, the number of families and the family sizes displayed significant differences in the genetic gains, with larger differences as generations progressed. The overall trend was that a greater number of families increase the gain in the long term.

### Diversity Loss

**FIGURE 4:**
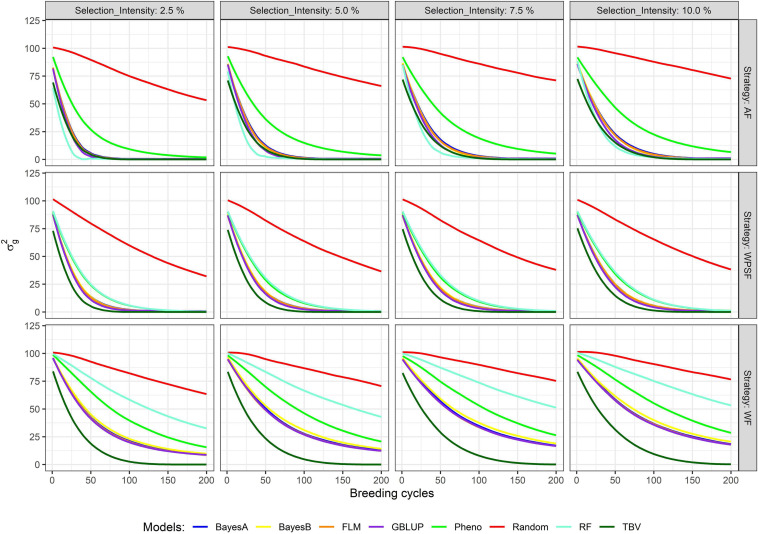
Genetic variance across 200 breeding cycles. Colors correspond to the different selection methods, grid columns represent the selection intensities (2.5, 5, 7.5, and 10%), and grid rows represent the breeding strategies, where individuals are selected across family (AF), within pre-selected families (WPSF), or within family (WF).

The decay in genetic variance overtime is presented in Figure 4. The number of cycles to exhaust 80% of the genetic variance is provided in Supplementary Table 3. The study simulates closed populations without the inflow of external variation, the existing genetic variance consumed overtime as selection takes place. Overall, a fast decay in genetic variance is observed under a higher selection pressure, whereas a lower selection pressure preserved more genetic variance in the long term. When selection was performed at random, over 80% of the initial genetic variance remained after 200 breeding cycles. The interaction between the selection intensity and selection strategy was significant (*p* < 0.01) across all selection models.

Within-family selection preserved the genetic variance for more cycles (Figure 4). Selection WF based on TBVs exhausted 80% of the genetic variance within 48–69 breeding cycles, whereas AF and WPSF selections on TBVs exhausted 80% of the diversity between 25 and 42 cycles (Supplementary Table 3). Depletion of genetic variance was more pronounced with GS. Under the selection intensity of 10%, BayesA selection WF exhausted 80% of the variance after 184 cycles, whereas selections AF and WPSF display the same diversity loss after 54 and 58 cycles, respectively.

**FIGURE 5:**
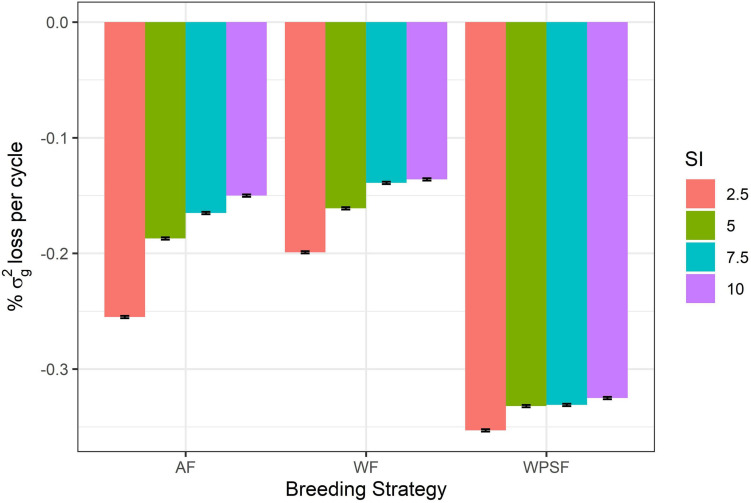
Genetic drift per cycle under random selection across family (AF), within pre-selected families (WPSF), or within family (WF) in different selection intensities (SI).

Diversity loss attributed to genetic drift is presented in Figure 5. These results assess the impact of bottlenecking the population through the various combinations of breeding strategy and selection intensity, utilizing random selections to avoid the confounding effect of directional selection. Higher rates of drift occurred under a higher selection pressure (2.5%). Strategy-wise, losses were highest for selection WPSF, with little difference across the selection intensities, ranging from −0.325 to −0.353%. The lowest rate of drift was observed under WF selection, with the rate of losses ranging from −0.199 to −0.136%.

## Discussion

Genomic prediction has become an important tool for selection and breeding in agriculture as it can enhance the rate of genetic gain in comparison to pedigree and phenotype-based selection by leveraging information on relationship and the linkage disequilibrium between the marker and the quantitative trait locus (QTL; Meuwissen et al., 2001; Habier and Fernando, 2009; Bernardo, 2010; Crossa et al., 2013, 2017; Daetwyler et al., 2013; de Los Campos et al., 2013). In soybean, the value of genomic prediction has been assessed and described in recent years (Jarquín et al., 2014; Xavier et al., 2016, 2018a,b; Diers et al., 2018; Matei et al., 2018; Xavier and Rainey, 2020). These studies agreed that adequate composition of the training data is imperative to successful and accurate prediction. The definition of an optimized training set entails (1) maximizing the genetic relationship between the training and target populations and (2) collecting phenotypic information from year–location combinations that represent the target population of environments. Whereas factors that affect genomic predictions for short-term gains have been well characterized, it is unclear which factors affect long-term genetic gains. The answer for that would come from long-term simulations, such as the present study. Primarily, simulations enable the optimization of the modern breeding program in animal and plant species (Yu et al., 2005; Hickey et al., 2014; Cowling et al., 2015, 2020; Gorjanc and Hickey, 2018; Muleta et al., 2018) by enabling the assessment of the breeding conditions that increase the rate of genetic gains, the conservation of useful genetic diversity, and the best allocation of breeding resource, such as the number of field plots, genotyping density, number of crosses, and population size (Heffner et al., 2010; Gonen et al., 2017; Gorjanc et al., 2017a,b).

Simulations indicate that linear models outperformed random forest for complex traits controlled by additive genetics and additive genotype-by-environment interactions. Under different scenarios, other studies found machine learning methods to display similar performances (Li et al., 2018; Ali et al., 2020). The discrepancy in the results is likely due to the nature of trait and population under evaluation, as machine learning predictions could be suitable for more structured populations and with some degree of epistatic control (Xavier, 2019; Abdollahi-Arpanahi et al., 2020). We also acknowledge that random forest was run with default settings in this study, and parameter tuning would benefit its predictive performance.

Selection factors provided a similar outcome to the findings in other studies (Gorjanc and Hickey, 2018; Santantonio and Robbins, 2020), where the authors assessed balancing short- and long-term sustainable gains in plant breeding. Their results indicate that higher population sizes provide higher long-term gains. An alternative framework for the maximization of long-term response to selection is proposed by Goddard (2009) based on the use of selection indexes that account for allele frequency aiming to account for the value of rare loci and in short- and long-term gains. Under limited resources, our simulations indicate that a lower selection pressure generally contributes to long-term gains at the cost of compromising short-term gains. Across breeding strategies, WPSF appears to provide reasonable gains in both the short and the long term while having the range of gains being less influenced by selection pressure. WPSF is an intermediate between AF and WF, and the results are, in fact, intermediary between the short-term gains provided by AF selections and the long-term gains provided by WF selection.

The real-life trend of genetic gains in soybeans is positive, but variable across geographies. In North America, the rates of genetic gain have been estimated to be 23.4 kg ha^–1^ year^–1^ (Fox et al., 2013), 26.5 kg ha^–1^ year^–1^ (Koester et al., 2014), and 16.8 kg ha^–1^ year^–1^ (Rogers et al., 2015). In the southern regions of Brazil, the rates of genetic gains were estimated to be 71.5 kg ha^–1^ year^–1^ (Lange and Federizzi, 2009) and 40.0 kg ha^–1^ year^–1^ (Todeschini et al., 2019); in Argentina, the rate has been reported to be 44.3 kg ha^–1^ year^–1^ (de Felipe et al., 2016). These reports provide insight from the perspective of traditional breeding progress before the deployment of GS and, in most cases, with lengthy breeding cycles with the choice of parents taking place in advanced generations and commercial products. Our simulations provided higher annual gains than what has been reported; however, with the advent of earlier evaluations and increasing trust in genomic prediction, it is likely that annual genetic gains will be progressively and iteratively optimized for multiple factors, including those evaluated in the present study (model, selection intensity, family size, and breeding strategy).

The selection of unproven parents from earlier generations is often interpreted as gambling with high risk and high rewards, even though much of the risk is mitigated with the use of genomic information with robust statistical models calibrated with phenotypic data from multiple years. In addition to advancements, more opportunities arise with the use of genomics to predict and select the best combinations for crossing that further increase the probability of generating elite offspring. Previous studies have evaluated population-level selection strategies in further detail (Bernardo, 2010; Jannink, 2010; Kemper et al., 2012; Daetwyler et al., 2015; Ma et al., 2016; Goiffon et al., 2017; Matei et al., 2018) with the goal of preserving the segregation of low-frequency haplotypes for long-term gains (Beukelaer et al., 2017). Balancing the number of families and the family size can be a fundamental part of the strategy to continue the steady gains overtime (Figure 2), and, whereas the difference is not perceived in the short term, the magnitude of grain increases significantly overtime. Yet, multiple factors should be taken into account when allocating resources in terms of the number of families and family size (Lindgren et al., 1997; Fu, 2015).

Scenarios simulated as provided herein were based on the parental selection at the F4 stage, which is commonly perceived as an early generation for recycling as the quality and the quantity of phenotypic data are still scarce, of doubtful quality, and in many cases, without replication. Nevertheless, early recycling is a promising framework for speeding up the rate of genetic gain by shortening the length of the breeding cycles. In fact, shortening the breeding cycles while inducing multiple cycles a year reproduces a framework referred to as “speed breeding” (Hickey et al., 2019; Nagatoshi and Fujita, 2019; Jähne et al., 2020). Recent studies often support recombination in the early stages of inbred development (Gaynor et al., 2017), more so as the accuracy of selection in the early stages benefits greatly from the GS. Another important aspect of parental selection regards the management of genetic diversity in modern plant breeding, which is largely ignored and not always adequately measured (Fu, 2015). Our results indicate that the multiple factors in the breeding design can affect the rate of diversity loss, mainly selection pressure and selection strategy (Supplementary Table 2), and that one must consider to balance these factors to attain the desired gain in the short term without compromising long-term gains. That is particularly the case for soybeans, whose germplasm-wise genetic diversity is considered low when compared to that of other species (Martin, 1982). Some Canadian soybean breeding programs have maintained diversity through decades of breeding while fixing maturity genes (Bruce et al., 2019). In the United States, soybean population structures and diversity varied by maturity group (Vaughn and Li, 2016), which suggests that new sources of variation could be obtained through the introgression of material from different regions.

The diversity available in breeding programs affects the accuracy of breeding values by dictating the amount of existing genetic signals to select upon an effective population size (Meuwissen, 2009). With restricted diversity, the genotyping density and marker distribution can be optimized to capture the existing variation in the target population with the goal of increasing genomic prediction accuracy (Ma et al., 2016). Of course, the long-term impacts of selection on genetic variance also vary depending on the genetic variance of interest, as the prominence of additive and non-additive variances is not the same over multiple cycles of selection (Paixão and Barton, 2016).

In soybeans, the management of diversity is necessary to ensure useful variability for future breeding objectives, such as yield performance under drought or waterlogging (Valliyodan et al., 2017), the seed oil and protein content profiles (Stewart-Brown et al., 2019), and disease resistance (de Azevedo Peixoto et al., 2017). Monitoring genetic diversity in the genomic era can be performed through tracking overtime changes in allele frequencies (Allier et al., 2019b; de Castro Lara et al., 2020; Meuwissen et al., 2020). We showed that selection could quickly exhaust genetic diversity under closed breeding systems, and breeding systems can benefit from balancing short gains to preserve diversity and assure long-term gains. Such balance had been the focal point of recent studies (Cowling et al., 2017; Gorjanc et al., 2018; Ru and Bernardo, 2019, 2020; Santantonio and Robbins, 2020) seeking for avenues to extend genetic resources with genomic tools, including the selection of material from germplasm collection to expend the genetic basis of elite programs. In addition to germplasm introgression, increases in genetic diversity in soybeans have been done in the past through mutagenic agents (Curtin et al., 2011; Khan, 2013; Haun et al., 2014; Demorest et al., 2016) and more recently, through genome editing techniques based on CRISPR-Cas9 (Cai et al., 2015, 2018a,b; Jacobs et al., 2015; Sun et al., 2015; Zheng et al., 2020) and target recombination for directional backcrossing (Ru and Bernardo, 2019, 2020).

The simulations performed in our study indicate that GS enables higher rates of genetic gain in the short and medium term compared with phenotype selection, but also led to faster extinction of the genetic variance. Thus, genomic prediction and selection must be applied mindfully with the purpose of maximizing gains while maintaining genetic variance. We found that a breeding strategy that balances selection at the family level, and within and across family at the individual level, can mitigate losses in genetic variance while providing satisfying genetic gains in the short term. Simulation is a powerful and inexpensive tool to test hypotheses, and for future studies, we envision addressing the importance of other important breeding parameters. Namely, future studies should focus on investigating (1) the optimal generation to select the parents and its trade-off with the accuracy of selection; (2) the influence of non-additive and non-infinitesimal genetic architecture and how machine learning would perform in such conditions; (3) the long-term effect of different models designed to select parental combinations; (4) the impact of different island models where new sources of variation are constantly infused into the main breeding panel; and (5) what would be the potential benefit of breeding hybrid soybeans assuming there are variable levels of dominance.

## Conclusion

Long-term gains were influenced by the interaction among GS models, breeding strategy, and selection intensity. Adequate handling of these factors will aid breeding programs to ensure genetic gains in short, medium, and long term. Therefore, the breeding strategy is the most influential factor and, therefore, is a key criterion to conserve genetic variance and obtain the highest population mean overtime. The absolute impact of the selection intensity is lower than that of the breeding strategy and GS model. The benefits of balancing family size and the number of families were not perceived on short-term gains. Additive GS models (BayesA, BayesB, FLM, and GBLUP) have similar behaviors in selecting the best individuals, whereas RF has poor predictive performance when implemented with default settings. In summary, a combination of strategies may be necessary for balancing the short-, medium-, and long-term genetic gains in breeding programs while preserving genetic variance.

## Data Availability Statement

The datasets presented in this study can be found in online repositories. The names of the repository/repositories and accession number(s) can be found below: https://github.com/Ederdbs/GenomicSelection.

## Author Contributions

ÉS and AX implemented the research, contributed with ideas to the algorithms, and wrote the manuscript. MF implemented the research, contributed ideas, and wrote the manuscript. All authors approved the final version to be published and agreed to be accountable for all aspects of the work in ensuring that questions related to the accuracy or integrity of any part of the work are appropriately investigated and resolved.

## Conflict of Interest

The authors declare that the research was conducted in the absence of any commercial or financial relationships that could be construed as a potential conflict of interest.

## Publisher’s Note

All claims expressed in this article are solely those of the authors and do not necessarily represent those of their affiliated organizations, or those of the publisher, the editors and the reviewers. Any product that may be evaluated in this article, or claim that may be made by its manufacturer, is not guaranteed or endorsed by the publisher.

## Footnotes

1
https://github.com/Ederdbs/GenomicSelection
