# Supplementary material for: Impact of Genomic Prediction Model, Selection Intensity, and Breeding Strategy on the Long-Term Genetic Gain and Genetic Erosion in Soybean Breeding
Source: Front Genet. 2021 Sep 1;12:637133. doi: 10.3389/fgene.2021.637133 (PMC8440908; doi:10.3389/fgene.2021.637133)
Supplement: Supplementary file 1 [file Data_Sheet_1.docx]

# SUPpLEMENTARYMATERIAL

## Population Mean

| **Supplementary Table 1** Estimated yield (t.ha^-1^) at cycles 10, 100 and 200 of selection across-family (AF), within pre-selected families (WPSF) or within-family (WF). | | | | | | | | | | | | | | | |
| --- | --- | --- | --- | --- | --- | --- | --- | --- | --- | --- | --- | --- | --- | --- | --- |
|  |  | 10 cycles | | | |  | 100 cycles | | | |  | 200 cycles | | | |
| Model | Strategy | Selection intensity | | | |  | Selection intensity | | | |  | Selection intensity | | | |
|  |  | 2.5% | 5.0% | 7.5% | 10.0% |  | 2.5% | 5.0% | 7.5% | 10.0% |  | 2.5% | 5.0% | 7.5% | 10.0% |
| BayesA | AF | 9.5 eA* | 8.9 eB | 8.5 dC | 8.2 eD |  | 25.0 kD | 28.1 gC | 29.2 eB | 29.8 cA |  | 25.0 mD | 28.5 oC | 30.5 kB | 32.1 gA |
|  | WPSF | 9.0 fA | 8.6 gB | 8.2 eC | 7.9 gD |  | 28.9 dB | 29.5 dA | 29.6 dA | 29.4 dA |  | 29.5 hD | 30.9 kC | 31.6 iB | 32.1 gA |
|  | WF | 7.8 jA | 7.3 kB | 6.9 jC | 6.8 kD |  | 28.9 dA | 27.8 hB | 26.3 jC | 25.7 jD |  | 34.1 eB | 34.3 fA | 33.9 eC | 33.5 eD |
| BayesB | AF | 9.4 eA | 8.7 fB | 8.4 dC | 8.1 fD |  | 23.1 mD | 26.1 kC | 27.6 iB | 28.5 fA |  | 23.1 pD | 26.2 qC | 28.1 oB | 29.9 kA |
|  | WPSF | 8.9 fA | 8.4 hB | 8.1 fC | 7.8 hD |  | 27.7 gC | 28.4 fB | 28.7 gA | 28.3 gB |  | 28.0 kD | 29.3 nC | 30.2 lB | 30.5 jA |
|  | WF | 7.5 kA | 7.1 lB | 6.8 kC | 6.6 lD |  | 27.2 hA | 26.2 kB | 24.8 lC | 24.2 lD |  | 32.2 gB | 32.6 jA | 32.0 hC | 31.6 hD |
| FLM | AF | 9.6 dA | 8.8 eB | 8.5 dC | 8.2 dD |  | 24.5 lD | 27.5 iC | 28.8 gB | 29.6 dA |  | 24.5 nD | 27.8 pC | 29.7 mB | 31.5 hA |
|  | WPSF | 9.0 fA | 8.6 gB | 8.1 fC | 7.8 hD |  | 28.6 eC | 29.4 dA | 29.4 dA | 29.1 eB |  | 29.1 iD | 30.5 lC | 31.2 jB | 31.7 hA |
|  | WF | 7.8 jA | 7.4 kB | 7.0 jC | 6.8 kD |  | 28.9 dA | 27.8 hB | 26.4 jC | 25.7 jD |  | 33.7 fB | 34.1 gA | 33.7 fB | 33.4 fC |
| GBLUP | AF | 9.6 dA | 9.0 dB | 8.5 dC | 8.3 dD |  | 22.1 nD | 24.8 mC | 26.5 jB | 27.7 iA |  | 22.1 qD | 24.8 rC | 26.6 pB | 28.3 mA |
|  | WPSF | 9.0 fA | 8.6 gB | 8.2 eC | 7.9 gD |  | 27.0 hC | 28.0 gB | 28.2 hA | 28.0 hB |  | 27.1 lD | 28.5 oC | 29.1 nB | 29.4 lA |
|  | WF | 7.8 jA | 7.4 kB | 7.0 jC | 6.9 kD |  | 27.9 fA | 27.2 jB | 25.7 kC | 25.2 kD |  | 32.1 gC | 32.8 iA | 32.5 gB | 32.2 gC |
| Pheno | AF | 8.2 gA | 7.7 jB | 7.4 iC | 7.1 jD |  | 28.0 fC | 29.0 eA | 29.0 fA | 28.7 fB |  | 32.0 gD | 35.0 eC | 36.3 dB | 36.7 dA |
|  | WPSF | 8.1 hA | 7.8 jB | 7.5 hC | 7.2 jD |  | 28.8 dB | 29.1 eA | 28.8 gB | 28.3 gC |  | 32.0 gC | 33.3 hB | 33.6 fA | 33.6 eA |
|  | WF | 6.3 mA | 6.1 mB | 5.8 lC | 5.7 mC |  | 25.5 iA | 24.5 nB | 22.9 mC | 22.2 mD |  | 35.3 dA | 35.2 dA | 33.9 eB | 33.2 fC |
| Random | AF | 3.6 oA | 3.6 oA | 3.6 nA | 3.6 oA |  | 3.6 qA | 3.6 qA | 3.6 pA | 3.6 pA |  | 3.6 sA | 3.6 uA | 3.6 sA | 3.6 pA |
|  | WPSF | 3.6 oA | 3.6 oA | 3.6 nA | 3.6 oA |  | 3.5 qA | 3.6 qA | 3.7 pA | 3.5 pA |  | 3.5 sA | 3.5 uA | 3.7 sA | 3.5 pA |
|  | WF | 3.6 oA | 3.6 oA | 3.6 nA | 3.6 oA |  | 3.6 qA | 3.6 qA | 3.6 pA | 3.6 pA |  | 3.6 sA | 3.6 uA | 3.5 sA | 3.6 pA |
| RF | AF | 8.0 iA | 7.9 iA | 7.7 gB | 7.4 iC |  | 12.8 pD | 16.6 oC | 19.4 nB | 21.6 nA |  | 12.8 rD | 16.9 tC | 20.0 rB | 22.6 nA |
|  | WPSF | 7.2 lA | 7.1 lB | 6.9 jC | 6.8 kD |  | 25.2 jB | 25.8 lA | 25.8 kA | 25.8 jA |  | 28.2 jD | 30.0 mC | 30.5 kB | 31.0 iA |
|  | WF | 5.1 nA | 5.0 nB | 4.8 mC | 4.8 nC |  | 16.0 oA | 14.8 pB | 13.4 oC | 12.9 oD |  | 23.5 oA | 22.2 sB | 20.3 qC | 19.7 oD |
| TBV | AF | 11.2 aA | 10.4 aB | 9.9 aC | 9.5 aD |  | 37.0 bC | 39.4 bB | 40.0 bA | 39.9 aA |  | 37.2 bD | 40.7 bC | 42.5 bB | 43.8 bA |
|  | WPSF | 10.7 bA | 10.0 bB | 9.5 bC | 8.9 bD |  | 35.7 cC | 36.3 cA | 36.0 cB | 35.4 bD |  | 35.8 cC | 37.1 cB | 37.6 cA | 37.7 cA |
|  | WF | 9.8 cA | 9.3 cB | 8.6 cC | 8.4 cD |  | 43.1 aA | 42.7 aB | 40.8 aC | 39.9 aD |  | 47.9 aC | 50.2 aB | 51.3 aA | 51.4 aA |
| * Lower case letters indicate difference in means across selection methods and breeding strategies (vertical comparison); Capital letters indicate difference in means across selection intensity within a same generation (horizontal comparison). Statistical test of multiple comparison based on Scott-Knott clustering at 5% of error probability. | | | | | | | | | | | | | | | |

## REGRESSION ANALYSIS BASED ON non-linear MODEL

We used non-linear regression, with model:

$$y=\theta_{a}\left( 1-e^{\left( -\theta_{v}x_{1} \right)} \right)+\theta_{d}x_{2}+\varepsilon,$$

Where: $y:$ is random variable analyzed in this case, population mean, $\theta_{a}:$ is parameter of maximum value, $\theta_{v}:$ is increase parameter, $\theta_{d}:$ is linear parameter of intensity increase, $x_{1}:$ is breeding cycle, $x_{2}:$is independent variable of selection intensity (SI), $\varepsilon:$ is random error. This models is reparametrized (Zeviani et al., 2012), and with parameter $\theta_{v}$, is possible created $\theta_{v}^{*}$ with relation:

$$\theta_{v}^{*}=-\frac{\log\left( 1-q \right)}{\theta_{v}},$$

where$q$(0 <$q$<1) represents proportion of maximum value, $\theta_{v}^{*}:$ represents breeding cycles that is equal $q$ define in this case in 0.8, this model is fitted in R software, using function nls.

| **Supplementary Table 2**Non-linear Regression parameters, for genetic gain select across-family (AF), within pre-selected families (WPSF) or within-family (WF). | | | | | | |
| --- | --- | --- | --- | --- | --- | --- |
| Models | Strategy | R² | $\theta_{a}$ (Max Gain) | $\theta_{d}$ (baseline SI) | $\theta_{v}$(Gain/cycle) | $\theta_{v}^{*}$(cycle to 80% total) |
| BayesA | AF | 0.949 | 26.732(±0.017)* | 0.439(±0.002) | 0.027(±0.001) | 58.964(±0.112) |
|  | WPSF | 0.984 | 30.894(±0.011) | 0.094(±0.001) | 0.026(±0.001) | 62.506(±0.064) |
|  | WF | 0.978 | 35.290(±0.018) | -0.137(±0.002) | 0.017(±0.001) | 96.612(±0.137) |
| BayesB | AF | 0.945 | 24.368(±0.016) | 0.460(±0.002) | 0.030(±0.001) | 53.391(±0.104) |
|  | WPSF | 0.981 | 29.341(±0.011) | 0.100(±0.001) | 0.027(±0.001) | 59.552(±0.064) |
|  | WF | 0.976 | 33.192(±0.017) | -0.113(±0.002) | 0.017(±0.001) | 96.558(±0.143) |
| FLM | AF | 0.949 | 26.021(±0.017) | 0.449(±0.002) | 0.028(±0.001) | 56.634(±0.107) |
|  | WPSF | 0.983 | 30.502(±0.011) | 0.091(±0.001) | 0.026(±0.001) | 61.184(±0.064) |
|  | WF | 0.979 | 34.977(±0.017) | -0.126(±0.002) | 0.017(±0.001) | 94.994(±0.132) |
| GBLUP | AF | 0.939 | 22.910(±0.015) | 0.462(±0.002) | 0.034(±0.001) | 47.585(±0.095) |
|  | WPSF | 0.978 | 28.315(±0.011) | 0.096(±0.001) | 0.030(±0.001) | 54.383(±0.062) |
|  | WF | 0.978 | 33.299(±0.016) | -0.096(±0.002) | 0.018(±0.001) | 90.843(±0.124) |
| Pheno | AF | 0.982 | 34.686(±0.017) | 0.201(±0.002) | 0.016(±0.001) | 100.310(±0.138) |
|  | WPSF | 0.988 | 33.448(±0.011) | 0.050(±0.001) | 0.019(±0.001) | 83.798(±0.083) |
|  | WF | 0.977 | 39.328(±0.036) | -0.126(±0.002) | 0.010(±0.001) | 156.934(±0.331) |
| RF | AF | 0.870 | 12.678(±0.018) | 0.881(±0.002) | 0.030(±0.001) | 53.520(±0.225) |
|  | WPSF | 0.987 | 29.809(±0.011) | 0.168(±0.001) | 0.017(±0.001) | 92.010(±0.101) |
|  | WF | 0.914 | 23.356(±0.034) | -0.116(±0.002) | 0.011(±0.001) | 142.523(±0.513) |
| TBV | AF | 0.973 | 40.415(±0.019) | 0.284(±0.002) | 0.024(±0.001) | 68.179(±0.097) |
|  | WPSF | 0.983 | 37.896(±0.013) | -0.021(±0.002) | 0.027(±0.001) | 59.384(±0.061) |
|  | WF | 0.990 | 54.048(±0.021) | -0.106(±0.002) | 0.015(±0.001) | 105.945(±0.115) |
| *Estimated parameter followed by standard error | | | | | | |

## Genetic variance

| **Supplementary Table 3**Number of breeding cycles necessary to exhaust 80% of genetic variance (E80GV); Estimated final population performance (Max) and the number of cycles to reach 80% of total genetic gains (NC80GG) considering prediction methods, selection intensity and the breeding strategy across-family (AF), within pre-selected families (WPSF) or within-family (WF) | | | | | | | | |
| --- | --- | --- | --- | --- | --- | --- | --- | --- |
| Model | Strategy | Number of cycles to E80GV | | | |  | Max  t.ha^-1^ | NC80GG |
|  |  | Selection Intensity | | | |  |  |  |
|  |  | 2.5% | 5.0% | 7.5% | 10.0% |  |  |  |
| BayesA | AF | 29 kD | 39 mC | 47 kB | 54 iA |  | 26 | 58 |
|  | WPSF | 41 iD | 47 kC | 52 iB | 58 hA |  | 30 | 62 |
|  | WF | 104 dD | 139 dC | 172 cB | 184 cA |  | 35 | 96 |
| BayesB | AF | 28 kD | 38 mC | 45 lB | 52 iA |  | 24 | 53 |
|  | WPSF | 41 iD | 47 kC | 53 iB | 57 hA |  | 29 | 59 |
|  | WF | 115 cD | 149 cC | 186 bB | 195 bA |  | 33 | 96 |
| FLM | AF | 28 kD | 38 mC | 45 lB | 52 iA |  | 26 | 56 |
|  | WPSF | 39 jD | 47 kC | 52 iB | 57 hA |  | 30 | 61 |
|  | WF | 101 eD | 136 eC | 168 dB | 179 dA |  | 34 | 94 |
| GBLUP | AF | 25 lD | 33 nC | 40 mB | 46 jA |  | 22 | 47 |
|  | WPSF | 37 jD | 44 lC | 49 jB | 53 iA |  | 28 | 54 |
|  | WF | 100 eD | 131 fC | 167 dB | 180 dA |  | 33 | 90 |
| Pheno | AF | 62 fD | 81 gC | 97 eB | 108 eA |  | 34 | 100 |
|  | WPSF | 57 gD | 64 iC | 68 gB | 75 fA |  | 33 | 83 |
|  | WF | 171 bC | 197 bB | 200 aA | 200 aA |  | 39 | 156 |
| Random | AF | 200 aA | 200 aA | 200 aA | 200 aA |  | - | - |
|  | WPSF | 200 aA | 200 aA | 200 aA | 200 aA |  | - | - |
|  | WF | 200 aA | 200 aA | 200 aA | 200 aA |  | - | - |
| RF | AF | 11 mD | 21 oC | 29 oB | 38 lA |  | 12 | 53 |
|  | WPSF | 57 gD | 67 hC | 72 fB | 76 fA |  | 29 | 92 |
|  | WF | 200 aA | 200 aA | 200 aA | 200 aA |  | 23 | 142 |
| TBV | AF | 25 lD | 32 nC | 37 nB | 42 kA |  | 40 | 68 |
|  | WPSF | 27 kD | 33 nC | 37 nB | 42 kA |  | 37 | 59 |
|  | WF | 48 hD | 55 jC | 66 hB | 69 gA |  | 54 | 105 |
| * Lower case letters indicate difference in means across selection methods and breeding strategies (vertical comparison); Capital letters indicate difference in means across selection intensity (horizontal comparison). Statistical test of multiple comparison based on Scott-Knott clustering at 5% of error probability. | | | | | | | | |

## Accuracy

The accuracy of genomic selection models is a key factor at many researches used for selection the model for used. Based on this result displayed inTable 5, we can see that the accuracy of models has interaction between breeding strategy and selection intensity, in long term. In BayesA for example, we can see in AF strategy that lowest selection intensity (2.5%), lost 80% of your variance with 87 breeding generation, and for 10% of selection intensity, this value is 154 breeding generation, it is very significative different. For this same model, BayesA, in strategy WF the results show that of end 200 breeding generation the 80% of accuracy is not loser. Similar conclusion can be extending for all Bayesian models, where for strategy WF, all never lost 80% of genetic variance, the strategy of WPSF and AF demonstrate similar behavior.

| 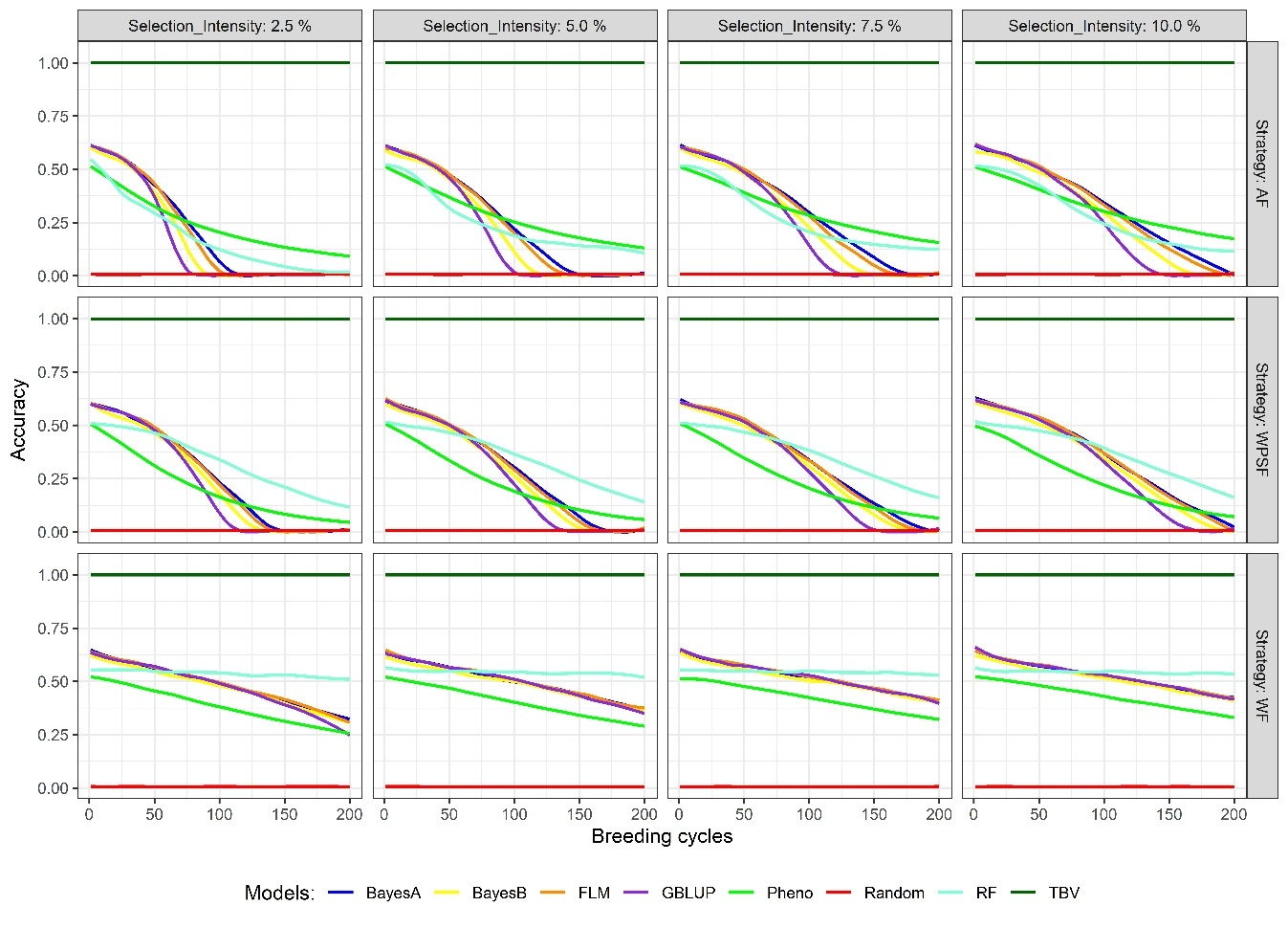 |
| --- |
| **Supplementary Figure 1**.Prediction accuracy across 200 breeding cycles. Colors correspond to different selection methods, grid columns represent selection intensity (2.5, 5, 7.5, 10%) and grid rows represent breeding strategies, were individuals are selected across-family (AF), within pre-selected families (WPSF) or within-family (WF). |
